# Gestational Diabetes Alters the Metabolomic Profile in 2nd Trimester Amniotic Fluid in a Sex-Specific Manner

**DOI:** 10.3390/ijms19092696

**Published:** 2018-09-10

**Authors:** Kathleen O’Neill, Jacqueline Alexander, Rikka Azuma, Rui Xiao, Nathaniel W. Snyder, Clementina A. Mesaros, Ian A. Blair, Sara E. Pinney

**Affiliations:** 1 Department of Obstetrics and Gynecology, Perelman School of Medicine, University of Pennsylvania, Philadelphia, PA 19104, USA; Katheen.O'Neill2@uphs.upenn.edu; 2 Center for Research in Reproduction and Women’s Health, Perelman School of Medicine, University of Pennsylvania, Philadelphia, PA 19104, USA; 3 Division of Endocrinology and Diabetes, The Children’s Hospital of Philadelphia, 3615 Civic Center Boulevard, Philadelphia, PA 19104, USA; Jacqueline.A.Alexander@gmail.com (J.A.); rlazuma@gmail.com (R.A.); 4 Department of Biostatistics, Perelman School of Medicine, University of Pennsylvania, Philadelphia, PA 19104, USA; rxiao@pennmedicine.upenn.edu; 5 AJ Drexel Autism Institute, Drexel University, Philadelphia, PA 19104, USA; nws28@drexel.edu; 6 Penn SRP Center and Center of Excellence in Environmental Toxicology, Perelman School of Medicine, University of Pennsylvania, Philadelphia, PA 19104, USA; c_mesaros@yahoo.com (C.A.M.); ianblair@upenn.edu (I.A.B.); 7 Department of Systems Pharmacology and Translational Therapeutics, Perelman School of Medicine, University of Pennsylvania, Philadelphia, PA 19104, USA; 8 Department of Pediatrics, Perelman School of Medicine, University of Pennsylvania, Philadelphia, PA 19104, USA

**Keywords:** gestational diabetes, metabolomics, fetal programming, sex specific effects, amniotic fluid

## Abstract

Maternal diabetes and obesity induce marked abnormalities in glucose homeostasis and insulin secretion in the fetus, and are linked to obesity, diabetes, and metabolic disease in the offspring, with specific metabolic characterization based on offspring sex. Gestational diabetes (GDM) has profound effects on the intrauterine milieu, which may reflect and/or modulate the function of the maternal–fetal unit. In order to characterize metabolic factors that affect offspring development, we profiled the metabolome of second trimester amniotic fluid (AF) from women who were subsequently diagnosed with gestational diabetes (GDM) using a targeted metabolomics approach, profiling 459 known biochemicals through gas chromatography/mass spectrometry (GC/MS) and liquid chromatography/mass spectrometry (LC/MS) assays. Using a nested case-control study design, we identified 69 total biochemicals altered by GDM exposure, while sex-specific analysis identified 44 and 58 metabolites in male and female offspring, respectively. The most significant changes were in glucose, amino acid, glutathione, fatty acid, sphingolipid, and bile acid metabolism with specific changes identified based on the offspring sex. Targeted isotope dilution LC/MS confirmatory assays measured significant changes in docosahexaenoic acid and arachidonic acid. We conclude that the sex-specific alterations in GDM maternal–fetal metabolism may begin to explain the sex-specific metabolic outcomes seen in offspring exposed to GDM in utero.

## 1. Introduction

Maternal diabetes and obesity induce marked abnormalities in glucose homeostasis and insulin secretion in the fetus, and are linked to obesity, diabetes, and metabolic disease in the offspring [1,2,3,4,5,6,7,8,9,10]. Gestational diabetes mellitus (GDM) has been shown to have profound effects on the intrauterine milieu, which may reflect and/or modulate altered function of the maternal–fetal unit [4]. Individuals whose cord blood or amniotic fluid insulin levels are elevated have a 3–4 fold risk for developing glucose intolerance, obesity, and type 2 diabetes in late childhood and as adults [5,11,12,13].

Identifying women who are at risk for development of GDM early in pregnancy has been stymied by a lack of sensitive and specific diagnostic tools. Currently, women are not treated for GDM until late in the second trimester, when a clinical diagnosis is made. Metabolomics has emerged as a technology that has the potential to both facilitate early detection of GDM and further our understanding of the pathogenesis and impact of the disease on both mothers with GDM and their offspring. Studies of the metabolome aim to provide an overall summary of the metabolic interactions within a given biological system [14], and allow for the simultaneous identification and quantification of a large number of analytes in a high-throughput and unbiased fashion [15]. Metabolomic investigations profile the downstream products of transcription, translation, and protein function. Thus, profiling the metabolome of second trimester amniotic fluid (AF) exposed to GDM may assist in the identification of biochemicals and pathways that are responsible for the early functional changes in the placenta and fetus exposed to GDM with greater sensitivity than transcriptomics or proteomics.

Previous studies employing metabolomics to study GDM have primarily focused on maternal blood or urine profiles, and although they have identified alterations in metabolic pathways including amino acid (AA), steroid hormone, glycerophospholipid, and fatty acid metabolism, these studies have been limited by inconsistent results [16]. Sampling amniotic fluid provides a direct measure of the metabolites of the fetal compartment compared to maternal blood and urine. This is especially meaningful in the period prior to 20 weeks of gestation, when the fetal skin is not yet keratinized, which allows for bi-directional diffusion of solute between the fetus and the amniotic fluid and the surfaces of the amnion, placenta, and umbilical cord [17]. Two previous studies profiled the amniotic fluid metabolome in women with GDM [18,19]. However, these studies were limited by a less sensitive analytic approach and failure to consider effects from critical biological variables, such as fetal sex, maternal age, and gestational age (GA) at delivery. Therefore, we hypothesized that isolation, identification, and quantification of metabolites present in second trimester AF from pregnancies subsequently diagnosed with GDM using sensitive mass spectrometry and nested case-control study design will provide new insights into how GDM alters maternal-placental-fetal metabolism, which ultimately results in permanent changes in developing fetal tissues and contributes to obesity and diabetes later in life.

## 2. Results

### 2.1. Clinical Characteristics of Women and Infants

We utilized an established biospecimen repository containing AF samples collected from women who underwent clinically indicated amniocentesis between 2002–2006. A nested case-control study design was employed by selecting 20 AF samples from mothers subsequently diagnosed with GDM (10 with female offspring and 10 with male offspring) and matched 1:1 with control AF samples without GDM. Matching criteria included maternal race and ethnicity, GA at amniocentesis, GA at birth, and fetal sex. Due to the matching algorithms used for the nested case-control design, there were no differences in maternal race, ethnicity, GA at time of amniocentesis, or GA at birth (Table 1). The vast majority of the samples were collected from white (>90%), non-Hispanic (≥95%) women. The mean age of all women was 37.4 ± 3.22 years, and the most common indication for amniocentesis was due to advanced maternal age. All samples studied were collected at a mean GA of 16.2 ± 0.56 weeks and the mean GA at birth was 39.0 ± 1.47 weeks. There were no statistical differences in infant birth weight.

### 2.2. Confirmation of GDM Classification

Initial GDM classification was based on participant report of GDM diagnosis in the postnatal outcome survey. C-peptide concentrations in AF were 4.4-fold higher in samples from women with GDM (*p* < 0.05), confirming reported GDM status (mean (pM) ± SD: GDM 224.3 ± 98.2; Control 50.6 ± 27.2). Insulin and c-peptide proteins are unable to cross the placenta and, thus, AF c-peptide concentration corresponds to fetal insulin production. Elevations in c-peptide levels in the amniotic fluid from women with GDM were observed in both male and female offspring (male fold change (FC) 5.8 (*p* < 0.05); female FC 3.6 (*p* < 0.05)).

### 2.3. Global Assessment of Metabolomic Data

**Figure 1:**
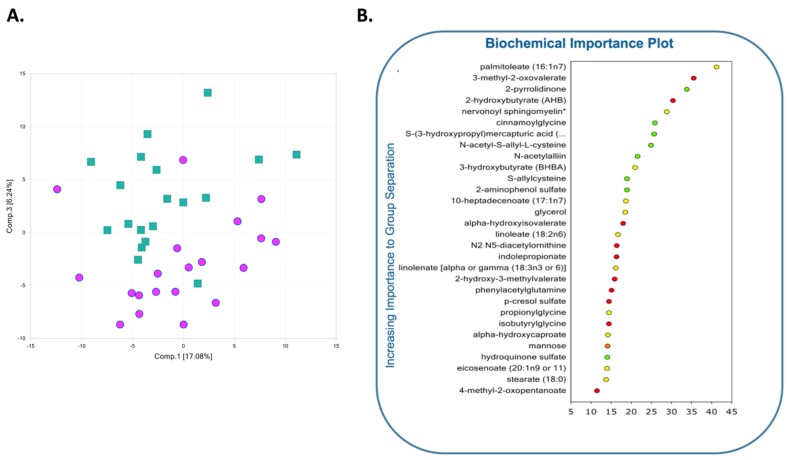
Global changes in metabolites in second trimester amniotic fluid (AF) from women with GDM. (**A**) Principal components analysis demonstrates that the global metabolome from second trimester GDM AF was overall distinguishable from AF in control non-diabetic pregnancies. GDM: green squares; Control: pink circles. (**B**) Random forest classification of amniotic fluid ranked metabolites by variable importance, and color-coded by biochemical class. Red: amino acid, orange: carbohydrate, yellow: lipid, green: xenobiotics.

Metabolomic analysis of second trimester AF from women with GDM (GDM AF) identified 69 total biochemicals (28 at higher concentrations, 41 at lower concentrations) with significantly altered quantities, using the threshold of *p* < 0.05. Separate male offspring analysis identified 44 altered biochemicals (7 present at higher concentrations, 37 present at lower concentrations) in GDM AF, while female offspring analysis identified altered 58 analytes (41 at higher concentrations, 17 at lower concentrations). Principal component analysis (PCA) showed GDM AF was generally distinguishable from control, despite some overlap, and led to a discernible shift of the AF metabolic profile (Figure 1A). In a separate unbiased and supervised classification analysis using the random forest (RF) approach, the AF profile differentiated the GDM and control groups with an overall predictive accuracy of 73% (Figure 1B) which is superior to random chance alone (50% accuracy), and is quite pronounced. Among the 30 top-ranking metabolites by RF analysis importance, biochemicals in AA, carbohydrates, and lipid pathways were identified as major contributors to the separation of groups. Despite a strong RF predictive accuracy, it is possible that the ranking of the top 30 metabolites may include some misclassification, since the predictive accuracy was not 100%, and this must be taken into account when interpreting the results.

For additional details, the full metabolomics metadata is available for review at https://upenn.box.com/s/o61ck21dbl67wcatvl73mv2sfh4lityr.

### 2.4. Metabolic Pathways Altered in AF Exposed to GDM

Among the 30 top-ranking metabolites resulting from the RF analysis, biochemicals spanning several different pathways (carbohydrates, AA, and lipids) were identified as contributing to separation of groups. Specific changes in biochemicals within these affected metabolic pathways are detailed below.

#### 2.4.1. Glucose Metabolism

As expected, the concentration of glucose was elevated (FC 1.2, *p* < 0.05) in AF samples from GDM mothers (Table 2). For pregnant women with GDM, glucose concentration in AF represents maternal plasma glucose transported across the placenta via glucose transporter proteins (GLUTs) 1, 3, 4, 8, 9, and 12 [20,21]. A study by Graca et al. reported a 14% increase in the concentration of glucose in second trimester AF of women who were later diagnosed with GDM, providing consistent evidence that mild glucose elevation is present in AF in the second trimester [19].

Other analytes involved in glucose metabolism were altered in midgestation GDM AF. 2-Hydroxybutyrate (AHB) concentrations were increased (FC 1.5, *p* < 0.05) while levels of 1,5-anhydroglucitol (1,5-AG) (FC 0.73, *p* < 0.05) and lactate were decreased in GDM. Increased AHB levels in GDM AF may be due to amino acid catabolism, activation of the glutathione stress pathway, or increased lipid peroxidation [22], and previous studies have shown activation of these pathways in women and fetuses exposed to GDM [23]. Additionally, increased AHB concentrations may indicate mitochondrial dysfunction as a result of ineffective utilization of propionyl-CoA (derived from α-ketobutyrate) in the tricarboxylic acid (TCA) cycle. 1,5-AG is structurally similar to glucose and, therefore, high serum glucose prevents 1,5-AG reabsorption in renal tubules, thus leading to 1,5-AG excretion in urine and lower serum levels. Decreased maternal serum levels of 1,5-AG indicates poor short-term glycemic control in both pregestational diabetes and GDM [24], and is associated with complications of diabetes in pregnancy, specifically neonatal hypoglycemia [24]. Although it is not known whether 1,5-AG crosses the placenta, if the inverse relationship between blood glucose and 1,5-AG persists in AF, decreased concentrations of 1,5-AG provide additional evidence for early AF glucose elevations in our GDM cohort. Finally, analyses stratified by offspring sex revealed increased concentration of lactate in GDM AF from male offspring (FC 1.28, *p* < 0.05). Lactate serves as a metabolic indicator of energy imbalance (the gap between energy expenditure and oxidative capacity).

#### 2.4.2. Amino Acid Metabolism

Overall, AA metabolites were significantly decreased in AF from women with GDM. Multiple amino acid metabolites, including glycine, glutamine, histidine, lysine, phenylalanine, tryptophan, and arginine were altered in GDM AF. AA metabolite reductions were more prominent in the male GDM AF samples (Table 3) with the most profound reductions in 3-methylglutarylcarnitine (lysine metabolite; FC 0.36, *p* < 0.05), indolepropionate (tryptophan metabolite; FC 0.26; *p* < 0.05) and *N*^2^,*N*^5^-diacetylornithine (arginine metabolite; FC 0.36; *p* < 0.05). 3-Methylglutarylcarnitine is produced by HMG-CoA lyase-mediated ketogenesis and leucine degradation. Reduced levels of 3-methylglutarylcarnitine may result from an inhibition of ketone production within the maternal–fetal unit. Indolepropionic acid is a gut microbial metabolite derived from tryptophan [25]. Increased serum levels of indolepropionic acid are associated with the reduced likelihood for progression to type 2 diabetes mellitus (T2DM) in overweight adults with impaired glucose tolerance [26]. *N*^2^,*N*^5^-diacetylornithine is an arginine metabolite, which is an essential AA for fetal and neonatal growth that also induces insulin secretion in pancreatic beta cells. However, little is known about the role of *N*^2^,*N*^5^-diacetylornithine in fetal metabolism and gestational diabetes.

By contrast, metabolites of tyrosine, leucine, and methionine were generally present at higher concentrations in AF from women with GDM, and many of these metabolites were profoundly increased in female offspring samples (Table 3). The metabolites with the largest increases were 3-(3-hydroxyphenyl) propionate (tyrosine metabolite), 3-(4-hydroxyphenyl) propionate (tyrosine metabolite), isovalerate (leucine metabolite), and 2-hydroxybutyrate (AHB) (methionine metabolite described above). 3,4-Hydroxyphenyl propionic acid has antioxidant, anti-inflammatory, and anticancer properties, but its role in GDM or fetal development has not been elucidated [27]. Previous studies assaying second trimester AF from women with GDM reported increased concentrations of 3-hydroxyisovalerate, a form of isovalerate which is associated with reduced biotin availability in AF from GDM pregnancies [18,19].

The addition of gamma-glutamyl residues to AA is an important mechanism for increasing translocation of AA across placental membranes, and plays a key role in the gamma-glutamyl cycle for maintaining glutathione homeostasis and redox status. Consistent reductions in gamma-glutamyl AAs were noted in GDM AF (particularly in female GDM AF) (Table 4), including marked reductions in gamma-glutamylglycine (FC 0.54), gamma-glutamylleucine (FC 0.59), gamma-glutamylphenylalanine (FC 0.55), gamma-glutamylthreonine (FC 0.6), gamma-glutamyltyrosine (FC 0.5), and gamma-glutamylvaline (FC 0.69) (all *p* < 0.05).

In summary, exposure to GDM, in utero, significantly alters the composition of AA and their metabolites in AF in midgestation. Given that AAs both serve as essential components for fetal growth and development, and mediate oxidative stress, these biochemical changes may contribute to phenotypic changes seen in offspring.

#### 2.4.3. Lipid Metabolism

Derangements in lipid handling and metabolism are central to diabetes metabolism. In women with GDM, increased concentrations of polyunsaturated fatty acids (PUFAs), including palmitoleate, stearate, eicosenoate, and long-chain saturated and monounsaturated fatty acids (LCFAs), including eicosapentaenoate, docosahexaenoate, linoleate, linolenate, dihomo-linolenate, arachidonate, and docosapentaenoate, were significantly increased in second trimester AF samples from GDM women (Table 5). Sex-specific analysis revealed decreases in medium chain fatty acids in AF from male offspring exposed to GDM (caproate, caprylate, and pelargonate) and increases in PUFAs and LCFAs in AF from female offspring exposed to GDM (palmitoleate, 10-heptadecenoate, stearate, eicosenoate, eicosapentaenoate, docosahexaenoate, linoleate, linolenate, dihomo-linolenate, arachidonate, and docosapentaenoate), with the most profound increases measured in palmitoleate and 10-heptadecenoate, each with over 5-fold increased concentrations compared to female control AF (FC 5.23 and 5.81, respectively (*p* < 0.05)).

**Figure 2:**
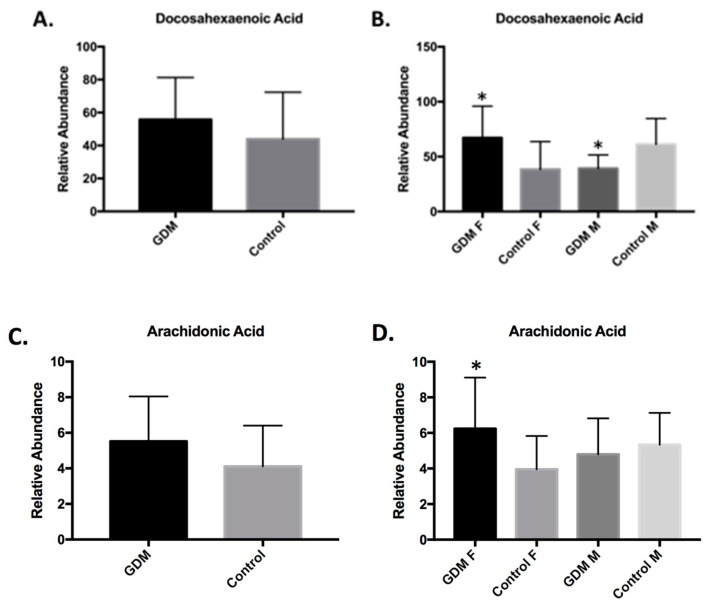
Docosahexaenoic acid (DHA) and arachidonic acid (AA) concentrations are increased in AF from female offspring exposed to GDM as measured by high resolution LC/MS analysis. (**A**) AF DHA relative abundance, all samples. (**B**) AF DHA relative abundance sex-specific analysis. (**C**) AF AA relative abundance, all samples. (**D**) AF AA relative abundance sex-specific analysis. * *p* < 0.05.

We conducted an additional confirmatory study using isotope dilution LC/high resolution MS to confirm the sex-specific elevations in docosahexaenoic acid and arachidonic acid in GDM AF from female offspring (Figure 2). The increased concentration of free fatty acids is accompanied by elevations in glycerol (FC 1.23; *p* < 0.05), suggesting that lipolysis is the source of free fatty acids. In addition, increased levels of the ketone bodies, acetoacetate and 3-hydroxybutyrate (BHBA), were measured (FC 1.55; *p* < 0.05). Ketones are produced from excess acetyl-CoA, and are byproducts of fatty acid oxidation used by peripheral tissues to meet energy demands. Both the changes in free fatty acids and elevations in glycerol and ketone bodies were more pronounced in the female GDM-exposed AF. Taken together, these findings suggest that early in second trimester in gestation, maternal insulin resistance in GDM triggers premature initiation of maternal adipose lipolysis, resulting in greater release of free fatty acids from maternal stores, which cross the placenta and enter AF.

#### 2.4.4. Sphingolipid Metabolism

Sphingomyelins are present in animal cell membranes, and the synthesis and degradation of sphingomyelin species produces signal transduction second messengers that regulate the innate immune response at the feto–maternal interface [28]. Additionally, disruptions in sphingomyelin content in plasma membranes and processing can have effects on insulin sensitivity [29]. In this cohort, pronounced elevations in several species of both saturated and unsaturated sphingomyelins (palmitoyl sphingomyelin (C16:0-SM), stearoyl sphingomyelin (C18:0-SM), nervonoyl sphingomyelin (C24:1-SM), and palmitoleoyl sphingomyelin (C16:1-SM)) were noted in GDM amniotic fluid (particularly in the female offspring group) (Table 6) (*p* < 0.05).

Taken together, these results suggest that changes in lipid metabolism reflected by increases in fatty acid, ketone, and sphingomyelin content in second trimester GDM AF demonstrate changes within fetus and/or placenta metabolism that may result in alterations in lipid processing, the innate immune response, and insulin sensitivity of the feto–maternal unit as early as the second trimester of pregnancy.

#### 2.4.5. Bile Acid Metabolism

Bile acids (BAs) play an important role in lipid metabolism and obesity. Elevated fetal BA levels have been linked to adverse pregnancy outcomes [30]. AF from GDM women carrying female offspring had moderate increases in several primary bile acids (BAs) in AF, specifically in glycocholate, chenodeoxycholate, and glycochenodeoxycholate (FC 1.36–1.64), but these increases did not reach statistical significance (Table 7). By contrast, the levels of secondary BAs taurolithocholate 3-sulfate, tauroursodeoxycholate, and glycohyocholate, were reduced (FC ~0.5 for all species, *p* < 0.05) in the GDM AF from male fetuses (Table 7). Since secondary bile acids result from interactions between intestinal bacteria and the gut, these results suggest the importance of interactions between the microbiome and the maternal–fetal interface in GDM.

## 3. Discussion

The characterization of the metabolome of second trimester AF from women subsequently diagnosed with GDM identifies significant changes in metabolic pathways involving glucose, AA, glutathione, fatty acid, sphingolipid, and bile acid metabolism, with specific changes identified based on offspring sex. Due to the use of a nested case-control study design to select matched pairs from a large repository of AF biospecimens, we were able to detect profound metabolic differences in GDM AF based on offspring sex. Although our sample size was small due to resource and sample constraints, we were able to enhance the confidence of the results of this exploratory analysis by matching GDM and control samples for criteria such as maternal age, GA at amniocentesis, GA at birth, and sex of the offspring. In addition, measurement of AF c-peptide concentrations reduced the risk of exposure (GDM) misclassification, a major strength of our study.

GDM AF from male offspring had the majority of significantly altered metabolites that were decreased (37/44), while GDM AF from female offspring had the majority of altered analytes that were increased (41/58). Many sex-associated differences in outcomes of the offspring have been observed in the setting of maternal diabetes, but mechanisms contributing to this phenomenon remain unknown. Pregnant women carrying a male fetus are at increased risk of developing GDM [31]. Additionally, maternal fasting blood glucose concentrations predicts adiposity in only male infants, while maternal body mass index (BMI) predicts adiposity in only female infants [32]. Male offspring of diabetic pregnancies are at higher risk of developing congenital malformations and respiratory disorders [33]. Recently, we reported that offspring sex affects DNA methylation and gene expression in term placentae from women with diabetes in pregnancy [34].

Stratified analyses revealed that all species of secondary bile acids (BAs) were lower in the GDM AF from male offspring. Secondary BAs are generated when microbiota in the gut deconjugate and dehydroxylate BA synthesized by the host. These findings strongly suggest that the maternal microbiome is altered by exposure to GDM, and importantly, the fetus is exposed to altered microbial metabolites. Our data indicate that fetal sex modulates this exposure either by altering the maternal microbiome or the microbial byproducts that are transported by the placenta. This fetal exposure is important for two reasons: (1) secondary BAs have membrane-destabilizing actions on the gut epithelium, and alterations in BAs have been associated with changes in glucose homeostasis in male offspring in a mouse model [35,36]; and (2) recent evidence suggests that the pregestational changes in maternal microbiota have an effect on the postnatal immune system of the offspring [37], and could contribute to the sex-specific effects detailed above.

Changes in free fatty acids and other lipid metabolites, branched chain AAs, sphingomyelins, and carbohydrate metabolism were either solely identified or particularly enriched in amniotic fluid from GDM women with female compared to male offspring. Specific lipid biomarkers in maternal serum have been proposed as a tool to screen for early detection of GDM [38]. In the analysis of LCFAs, concentrations of palmitoleate (C16:1n7) were enriched 5-fold in GDM AF from female offspring, while no significant changes were detected in GDM AF from male offspring. Palmitoleate is product of de novo lipogenesis that enhances insulin sensitivity in adipose tissue and liver, although, its function in fetal liver, placenta, or fetal adipocytes has not been characterized [39,40]. In addition, both the targeted metabolomic analysis and the high-resolution LC/MS assays confirmed that AF from female offspring with GDM had significantly increased concentrations of docosahexanoic acid (DHA; 22:6n3) and arachidonic acid (AA; 20:3n6), metabolites of the essential fatty acids linolenic acid (20:3n3) and linoleic acid (18:2n6) that are derived from maternal dietary courses. Given the relationship between palmitoleate and enhanced insulin sensitivity, and the critical roles DHA and AA play in fetal neural development and immune function, palmitoleate, DHA, and AA enrichment in GDM-exposed female AF samples may represent a protective response to the malprogramming effects from GDM exposure that are absent in male offspring. In fact, in a recent study that aimed to describe the sexual dimorphism of in utero programming effects, the relationship between GDM exposure, in utero, and childhood adiposity (age 6–8 years), was only significant for male offspring after adjusting for offspring sex [41].

Not all the metabolic pathways that were altered by in utero GDM exposure varied by sex of the offspring. The significant reductions in gamma-glutamyl AA pathway metabolites were not affected by offspring sex. Once inside the cell, gamma-glutamyl AAs serve as substrates for glutathione synthesis [42], an essential cofactor for antioxidant and detoxifying enzymes. Decreased ability to generate gamma-glutamyl AAs could result in insufficient glutathione production and a reduced ability of the fetus to neutralize the increased placental oxidative stress that accompanies GDM [43]. Alternatively, reduced levels of gamma-glutamyl AAs in the GDM AF could suggest increased conversion of these substrates by gamma-glutamyl transferase (GGT) to glutathione. Regardless, if the reduction in gamma-glutamyl AA concentration is due to deficient production or increased utilization, these findings provide evidence that GDM is accompanied by changes in the glutathione pathway [16,43]. Alterations in glutathione metabolism could also modulate the known relationship between hyperglycemia, hyperinsulinism, insulin resistance, and the increased ROS and oxidative stress observed in GDM [16].

GDM exposure resulted in increased levels of ketones in AF, suggesting that enhanced beta-oxidation of fatty acids originated from either maternal or placental sources with no differences detected based on offspring sex. In normal healthy pregnancies, the third trimester coincides with increased supply of fatty acids, glycerol, and ketone bodies to the fetus to meet energy demands for accelerated growth and adipose deposition. However, the increased levels of fatty acids, glycerol, and ketone bodies in second trimester AF from the GDM pregnancies suggests that these processes may be accelerated. Perturbations in the availability of fatty acids, glycerol, and ketone bodies, as fetal fuel sources in second trimester, have the potential to negatively impact fetal development by increasing oxidative stress and lipid peroxidation. Indeed, consistent elevations in monohydroxylated fatty acids, byproducts from an oxidative environment, were noted in AF from women with GDM carrying female offspring, and provide further support that these individuals may be at an increased risk for altered lipid metabolism during gestation.

The findings presented, here, confirm many of the metabolic changes reported by two earlier studies of second trimester AF exposed to GDM by Diaz et al. and Graca et al., including elevations in isovalerate and 2-hydroxybutyrate in AF from GDM women [18,19]. However, we were able to detect a much larger number of pathways and metabolites in AF altered by GDM exposure and sex-specific differences due, in part, to the more sensitive and specific methodology employed. The use of tandem mass spectrometry for targeted analyte quantification enabled the successful identification of over 450 known compounds. Other metabolomic studies in serum from women with GDM have reported increases in 3-hydroxybutyrate, lactate and glycerol, alanine, proline, glutamine/glutamate, arginine, leucine/isoleucine, asparagine/aspartate, and branched chain AAs [44,45,46].

Our findings are limited by the fact that we were only able to profile the AF metabolome at one timepoint during midgestation, and a study with repeated measures would have provided a better assessment of the AF metabolome in GDM, but is not feasible with human samples. Furthermore, no biochemical comparisons had a false discovery rate (*q*-value) <0.05. Although a higher *q*-value diminishes confidence, it does not necessarily rule out the significance of a result. Increasing the number of individuals sampled would help increase power; however, this would be difficult given the associated cost and fact that invasive prenatal AF testing is at historic lows, due to increased use of non-invasive prenatal testing [47]. We have attempted to increase our confidence by confirming the findings detected in unbiased metabolomic analyses using complementary methods (mass spectrometry); given the limited power and higher false discovery rates (FDRs), these findings must be interpreted with caution. An additional limitation is the inability to study the effect of maternal BMI as an independent variable, since these data were not available for the cohort. Finally, since amniocentesis is a rare medical procedure, given the availability of cell free fetal DNA testing for cytogenetic studies, we were unable to validate our findings in a separate cohort.

## 4. Materials and Methods

### 4.1. Amniotic Fluid Samples

Women with healthy singleton term pregnancies without a prior history of diabetes, other pregnancy complications, or known fetal anomalies, were selected for study. AF was collected in accordance with a strict research protocol at GA 16–18 weeks, and stored in polypropylene cryogenic vials at −80 °C. Clinical charts were abstracted for maternal age, race, and ethnicity, GA at amniocentesis, indication for amniocentesis, cytogenetic results, pregnancy outcome data (i.e., birth weight and GA at birth), and maternal health history, including pregnancy complications. Post-birth outcome surveys were administered one month after delivery, during which GDM status in pregnancy was captured. Women with multiple gestations or whose infants had chromosomal abnormalities, birth defects, prematurity, or maternal complications other than GDM, were excluded. Written informed consent was obtained prior to amniocentesis. This study was approved by the Children’s Hospital of Philadelphia and University of Pennsylvania Institutional Review Boards (11-8050, 6 October 2011).

A nested case-control study design was used by selecting 20 AF samples from mothers subsequently diagnosed with GDM (10 with female offspring and 10 with male offspring) and matched 1:1 with control AF samples without GDM. Matching criteria included maternal race and ethnicity, GA at amniocentesis, GA at birth, and fetal sex. Data on maternal BMI, maternal hemoglobin A1C, or treatment modality for GDM, were not collected.

### 4.2. C-Peptide Measurement

C-peptide was measured in AF samples to confirm GDM classification with ELISA (Mercodia; Uppsala, Sweden) and normalized to total protein concentration (BSA assay; Pierce; Waltham, MA, USA).

### 4.3. Targeted Metabolomic Analysis

Targeted metabolomic analysis of 459 named biochemicals in 100 µL aliquots of AF was performed by Metabolon Inc., (Durham, NC, USA). Analytes were extracted and prepared using a standard solvent extraction method [48]. The extracted samples were split into equal parts for analysis on complementary gas chromatography (GC) (mass spectrometry (MS) (Thermo-Finnigan Trace DSQ fast-scanning single-quadrupole mass spectrometer (Thermo Scientific, Waltham, Mass, USA), and liquid chromatography (LC)/MS platforms (Waters ACQUITY ultra-performance liquid chromatography (UPLC) (Waters Corp, Milford, Mass, USA) and a Thermo Scientific Q-Exactive high resolution/accurate mass spectrometer (Thermo Scientific, Waltham, Mass, USA) [48]. Welch’s unequal variances two-sample t-test, and ANOVA, were used to identify biochemicals that differed significantly between groups. Two-way ANOVA was used to identify biochemicals exhibiting significant interaction and main effects of GDM. FDR was used to account for multiple comparisons [49,50]. Principal component analysis, and unbiased and supervised classification analysis using a random forest (RF), were performed [49]. Please see Supplementary Methods for additional details.

### 4.4. Confirmatory PUFA Analysis by Isotope Dilution LC/High Resolution MS

Non-esterified polyunsaturated fatty acids (PUFAs) were analyzed by liquid chromatography-high resolution mass spectrometry after precipitation of proteins in acetonitrile. For AF, specific gravity was measured by a Reichert clinical refractometer (Reichart, Buffalo, NY, USA), after dilution by a factor of 3 in water. For relative levels of PUFAs, 20 µL of AF was extracted by addition of 20 µL of a mix of stable isotope-labeled internal standards (ALA-d8, AA-d8, DGLA-d6, DHA-d5, DPA-d5, EPA-d5, and LA-d4), followed by addition of 60 µL of 4 °C acetonitrile with 0.1% BHT, and then vortex-mixing for 30 s. After acetonitrile precipitation and mixing, samples were centrifuged at 16,000*g* for 5 min at 4 °C, and the supernatant was transferred to a 96-well plate, evaporated to dryness under nitrogen gas, then resuspended in 100 µL 5% acetonitrile in water. Of each sample, 10 µL was injected on a Ultimate 3000 UHPLC with a Waters XBridge C18 column (2.1 × 150 mm, 3.5 µm) (Waters Corp., Milford, CT, USA) using a reversed phase gradient with solvent A, of 0.2 mM ammonium fluoride in water, to solvent B, of methanol, at 0.2 mL/min flow rate.

## 5. Conclusions

In conclusion, targeted metabolomic analysis of second trimester AF from women subsequently diagnosed with GDM revealed profound changes in glucose, AA, glutathione, fatty acid, bile acid, and sphingolipid metabolism. Many of the metabolic pathways that were altered by in utero exposure to GDM were affected by offspring sex. Our findings should be interpreted as representing a combination of maternal, fetal, and placental metabolism which, at midgestation, are functioning as an integrated single unit. While these women had no history of prior diagnoses of diabetes or GDM, our data indicate profound differences in fetal metabolism early in the second trimester before most women are even tested for GDM. The alterations in the GDM maternal-fetal metabolism described above, may begin to explain the sex specific metabolic changes seen in offspring exposed to GDM in utero.

## Figures and Tables

**Table 1:** Maternal and infant characteristics.

Maternal and Infant Characteristics	GDM *n* = 20	Control *n* = 20	*p* Value
Maternal Age (years, mean ± SD)	37.4 ± 3.6	37.3 ± 2.8	NS
Gestational Age at Amniocentesis (weeks, mean ± SD)	16.2 ± 0.6	16.2 ± 0.5	NS
Gestational Age at Birth (weeks, mean ± SD)	39.1 ± 1.4	38.9 ± 1.6	NS
Birthweight (grams, mean ± SD)			
All Offspring Female Offspring Male Offspring	3389 ± 526 3079 ± 411 3733 ± 422	3419 ± 480 3345 ± 553 3493 ± 410	0.83 0.16 0.08

NS, not significant. * *p* < 0.05. GDM: gestational diabetes; SD: standard deviation.

**Table 2:** Changes in glucose metabolism in amniotic fluid from women with GDM.

Glucose Metabolism	GDM		
	Control		
	All	Female	Male
1,5-anhydroglucitol (1,5-AG)	0.73	0.86	0.61
glucose	1.19	1.30	1.07
pyruvate	0.82	0.71	1.00
lactate	1.11	0.95	1.28
glycerate	1.10	1.36	0.92
2-hydroxybutyrate (AHB)	1.50	1.52	1.47

Normalized values expressed as fold change. Green: indicates significant difference (*p* ≤ 0.05) between the groups shown, metabolite ratio of <1.00. Red: indicates significant difference (*p* ≤ 0.05) between the groups shown; metabolite ratio of ≥1.00. Non-colored cell: mean values are not significantly different for that comparison. GDM: gestational diabetes.

**Table 3:** Changes in amino acid metabolism in second trimester AF of women with GDM.

Amino Acid Metabolis	Amino Acid	GDM		
		Control		
		All	Female	Male
Glycine Metabolism	glycine	0.81	0.81	0.81
	betaine	0.89	0.92	0.87
	serine	0.86	1.04	0.67
Glutamate Metabolism	glutamate	0.92	1.02	0.84
	pyroglutamine	0.68	0.85	0.56
Histidine Metabolism	histidine	0.84	0.88	0.79
	*N*-acetyl-1-methylhistidine	0.86	0.7	1.03
Lysine Metabolism	glutarylcarnitine (C5)	0.83 *	0.86	0.80
	3-methylglutarylcarnitine	0.36 *	0.40	0.33
Phenylalanine Metabolism	phenylacetylglutamine	0.72 *	0.77	0.68
Tyrosine Metabolism	*p*-cresol sulfate	0.75 *	0.79	0.71
	3-(3-hydroxyphenyl)- propionate	1.53	0.91	2.71
	3-(4-hydroxyphenyl)- propionate	1.25	0.54	5.3
	3-phenylpropionate- (hydrocinnamate)	0.48	0.39	0.59
	*p*-cresol-glucuronide	0.61	0.49	0.75
Tryptophan Metabolism	indolepropionate	0.26	0.12	0.82
	*N*-acetylkynurenine	0.87	0.57	1.08
Leucine Metabolism	4-methyl-2-oxopentanoate	1.15	1.19	1.11
	isovalerate	1.33	1.94	0.95
	isovalerylglycine	0.61	0.53	0.73
	alpha-hydroxyisovalerate	1.19	1.13	1.25
	3-methyl-2-oxovalerate	1.28	1.29	1.26
	2-hydroxy-3-methylvalerate	1.31	1.26	1.35
	isobutyrylglycine	0.59	0.54	0.67
Methionine Metabolism	methionine sulfoxide	1.11	1.49	0.82
	2-aminobutyrate	1.14	1.26	1.01
	2-hydroxybutyrate (AHB)	1.5	1.52	1.47
	cysteine	0.92	0.99	0.84
Arginine Metabolism	arginine	0.91	0.97	0.84
	urea	0.81	0.81	0.81
	ornithine	0.90	0.96	0.84
	citrulline	0.91	1.08	0.75
	*N*^2^,*N*^5^-diacetylornithine	0.36	0.44	0.28
	*N*-acetylcitrulline	0.49	0.51	0.46
Polyamine Metabolism	5-methylthioadenosine (MTA)	0.83	0.93	0.74

Normalized values expressed as fold change. Green: indicates significant difference (*p* ≤ 0.05) between the groups shown, metabolite ratio of <1.00. Light green: narrowly missed statistical cutoff for significance 0.05 < *p* < 0.10, metabolite ratio of <1.00. Red: indicates significant difference (*p* ≤ 0.05) between the groups shown; metabolite ratio of ≥1.00. Light red: narrowly missed statistical cutoff for significance 0.05 < *p* < 0.10, metabolite ratio of ≥1.00. Non-colored cell: mean values are not significantly different for that comparison. GDM: gestational diabetes.

**Table 4:** Decreased gamma-glutamyl amino acids in amniotic fluid of women with GDM.

Gamma-Glutamyl Amino Acid	GDM		
	Control		
	All	Female	Male
gamma-glutamylalanine	0.81	0.16	1.88
gamma-glutamylglutamate	0.86	0.23	1.67
gamma-glutamylglycine	0.55	0.26	0.84
gamma-glutamylisoleucine	0.78	0.87	0.69
gamma-glutamylleucine	0.59	0.63	0.54
gamma-glutamyllysine	0.51	0.18	1.29
gamma-glutamylmethionine	0.79	0.19	1.75
gamma-glutamylphenylalanine	0.55	0.54	0.57
gamma-glutamylthreonine	0.6	0.66	0.55
gamma-glutamyltyrosine	0.5	0.48	0.53
gamma-glutamylvaline	0.69	0.72	0.67

Normalized values expressed as fold change. Green: indicates significant difference (*p* ≤ 0.05) between the groups shown, metabolite ratio of <1.00. Light green: narrowly missed statistical cutoff for significance 0.05 < *p* < 0.10, metabolite ratio of <1.00. Non-colored cell: mean values are not significantly different for that comparison. GDM: gestational diabetes.

**Table 5:** Fatty acid subtypes in second trimester AF from women with GDM.

Fatty Acid Subtype	Fatty Acid Species	GDM		
		Control		
		All	Female	Male
Medium Chain Fatty Acids	caproate (6:0)	0.93	1.08	0.8
	heptanoate (7:0)	0.99	1.06	0.94
	caprylate (8:0)	1.13	2.02	0.62
	pelargonate (9:0)	0.94	1.09	0.81
	caprate (10:0)	1.04	1.08	1.01
	10-undecenoate (11:1n1)	0.86	1.30	0.53
Long Chain Fatty Acids	palmitoleate (16:1n7)	3.32	5.23	1.37
	10-heptadecenoate (17:1n7)	3.44	5.81	1.14
	stearate (18:0)	1.19	1.4	1.02
	arachidate (20:0)	1.05	1.22	0.90
	eicosenoate (20:1n9 or 11)	1.88	2.82	1.15
Polyunsaturated Fatty Acids (n3 and n6)	eicosapentaenoate (EPA; 20:5n3)	1.46	1.99	1.07
	docosapentaenoate (DPA; 22:5n3)	1.49	1.76	1.18
	docosahexaenoate (DHA; 22:6n3)	1.45	2.29	0.90
	linoleate (18:2n6)	1.73	2.66	1.12
	linolenate (alpha or gamma; (18:3n3 or 6))	2.05	3.47	1.22
	dihomo-linolenate (20:3n3 or n6)	1.97	3.1	1.30
	arachidonate (20:4n6)	1.61	2.13	1.23
	docosapentaenoate (n6 DPA; 22:5n6)	1.45	1.98	1.02
	dihomo-linoleate (20:2n6)	1.26	1.52	1.08

Normalized values expressed as fold change. Green: indicates significant difference (*p* ≤ 0.05) between the groups shown, metabolite ratio of <1.00. Red: indicates significant difference (*p* ≤ 0.05) between the groups shown; metabolite ratio of ≥1.00. Light red: narrowly missed statistical cutoff for significance 0.05 < *p* < 0.10, metabolite ratio of ≥1.00. Non-colored cell: mean values are not significantly different for that comparison. GDM: gestational diabetes.

**Table 6:** Sphingolipid species in second trimester AF of women with GDM.

Sphingolipid Subtypes	GDM		
	Control		
	All	Female	Male
palmitoyl sphingomyelin (C16:0-SM)	1.21	1.39	1.05
stearoyl sphingomyelin (C18:0-SM)	1.21	1.51	1.00
nervonoyl sphingomyelin (C24:1-SM)	1.21	1.40	1.03
palmitoleoyl sphingomyelin (C16:1-SM)	1.26	1.65	0.96

Normalized values expressed as fold change. Red: indicates significant difference (*p* ≤ 0.05) between the groups shown; metabolite ratio of ≥1.00. Light red: narrowly missed statistical cutoff for significance 0.05 < *p* < 0.10, metabolite ratio of ≥1.00. Non-colored cell: mean values are not significantly different for that comparison. GDM: gestational diabetes.

**Table 7:** Changes in bile acid metabolism in second trimester AF of women with GDM.

Bile Acid Subtype	Bile Acid Species	GDM		
		Control		
		All	Female	Male
Primary Bile Acid Metabolism	cholate	0.9	0.77	1.04
	glycocholate	1.31	1.47	1.01
	taurocholate	1.02	1.23	0.76
	chenodeoxycholate	1.3	1.64	1.02
	glycochenodeoxycholate	1.29	1.36	1.17
	taurochenodeoxycholate	1.11	1.14	1.04
	tauro-beta-muricholate	0.98	1.01	0.95
Secondary Bile Acid Metabolism	deoxycholate	1.03	0.99	1.08
	glycolithocholate sulfate	0.58	0.79	0.46
	taurolithocholate 3-sulfate	0.72	1.02	0.57
	glycoursodeoxycholate	0.99	0.7	3.26
	tauroursodeoxycholate	0.82	1.02	0.58
	glycohyocholate	1.29	1.98	0.62
	glycocholenate sulfate	1.06	1.47	0.83
	taurocholenate sulfate	0.9	1.38	0.66

Normalized values expressed as fold change. Green: indicates significant difference (*p* ≤ 0.05) between the groups shown, metabolite ratio of <1.00. Light green: narrowly missed statistical cutoff for significance 0.05 < *p* < 0.10, metabolite ratio of <1.00. Light red: narrowly missed statistical cutoff for significance 0.05 < *p* < 0.10, metabolite ratio of ≥1.00. Non-colored cell: mean values are not significantly different for that comparison. GDM: gestational diabetes.

## Supplementary Materials

Supplementary materials can be found at http://www.mdpi.com/1422-0067/19/9/2696/s1.

## Abbreviations

1,5-AG	1,5-anhydroglucitol
AA	amino acid
AF	amniotic fluid
AHB	2-hydroxybutyrate
ANOVA	analysis of variance
BA	bile acids
BMI	body mass index
DOAJ	directory of open access journals
FC	fold change
GA	gestational age
GLUT	glucose transporter protein
GC/MS	gas chromatography/mass spectrometry
GDM	gestational diabetes mellitus
LC/MS	liquid chromatography/mass spectrometry
MDPI	Multidisciplinary Digital Publishing Institute
PCA	principal component analysis
RF	random forest
T2DM	type 2 diabetes mellitus
TCA	tricarboxylic acid
